# Assessment of ARX expression, a novel biomarker for metastatic risk in pancreatic neuroendocrine tumors, in endoscopic ultrasound fine‐needle aspiration

**DOI:** 10.1002/dc.24368

**Published:** 2019-12-17

**Authors:** Wenzel M. Hackeng, Folkert H. M. Morsink, Leon M. G. Moons, Christopher M. Heaphy, G. Johan A. Offerhaus, Koen M. A. Dreijerink, Lodewijk A. A. Brosens

**Affiliations:** ^1^ Department of Pathology University Medical Center Utrecht Utrecht The Netherlands; ^2^ Department of Gastroenterology University Medical Center Utrecht Utrecht The Netherlands; ^3^ Department of Pathology Johns Hopkins Medical Institutions Baltimore Maryland; ^4^ Department of Endocrinology and Internal Medicine Amsterdam University Medical Center Amsterdam The Netherlands

**Keywords:** cytology, endoscopic ultrasound, neuroendocrine tumor, pancreas, prognostic markers

## Abstract

**Background:**

The transcription factors ARX and PDX1, and alternative lengthening of telomeres (ALT) were recently described as prognostic markers for resected non‐functional pancreatic neuroendocrine tumors (PanNETs). ALT positive tumors with ARX expression relapse most often. Currently, tumor size is the only preoperative marker used to decide whether or not to operate, thus additional preoperative prognostic markers are needed. Therefore, it is critical to assess the performance of these biomarkers on preoperative cytologic specimens.

**Methods:**

Endoscopic fine‐needle aspiration cellblock material and corresponding surgical specimens of 13 patients with PanNETs were assessed for histology, immunohistochemical staining of ARX, PDX1, Synaptophysin, Ki67, and telomere‐specific fluorescence in situ hybridization to detect ALT, and then associated with clinicopathological features. Scoring for ARX and PDX1 was performed blinded by two independent observers.

**Results:**

Of the 13 surgical specimens, 8 were ARX+/PDX1−, 2 ARX−/PDX1+, and 3 ARX+/PDX1+. Concordance between cytologic and surgical specimens for ARX protein expression was 100%, whereas concordance for PDX1, ALT, and WHO tumor grade was 85%, 91%, and 73%, respectively. There was a perfect inter‐observer agreement in ARX and PDX1 scoring.

**Conclusion:**

ARX can reliably be determined in cytologic specimens and has low inter‐observer variability. For cytology, false‐positive PDX1 expression was observed, possibly due to contamination or sampling, while ALT had a false‐negative case due to incomplete sampling. As previously observed, tumor grade is underestimated in cytologic specimens. Thus, ARX and ALT are the most promising markers to predict metastatic behavior in PanNETs, thereby warranting further validation in larger studies.

## 1 INTRODUCTION

Neuroendocrine tumors of the pancreas (PanNET) are the second most common malignancy of the pancreas. Hepatic metastases are the leading cause of death in these patients and almost half have metastatic liver involvement at first presentation.^1^ However, for the other half, preventing liver metastases is the primary goal for treatment and follow‐up, in particular for non‐functional cases which comprise the largest proportion of PanNETs. Surgical resection of the primary tumor can reduce the risk of metastasis, but is associated with significant morbidity and mortality. Importantly, surgery may be unnecessary in some cases, as a subgroup of PanNETs is indolent and does not give metastases. The choice for surgery—a trade‐off between the associated risks of surgery, postsurgical morbidity and the potential benefit of preventing metastasis—is difficult, as reliable preoperative prediction of indolent or aggressive biology is lacking. Most established prognostic factors for development of metastasis, such as microscopic invasiveness, lymph node involvement, perineural invasion, and tumor grade can only reliably be determined after surgery. The only marker with prognostic value that can easily be preoperatively assessed is tumor size, based on CT‐ or MR‐imaging or endoscopic ultrasound, which is used as the key criterion to decide on surgery in non‐functional PanNETs in European and American guidelines.^2^, ^3^ Thus, additional markers for preoperative risk stratification are urgently needed.

Larger tumor size is an independent predictor of disease relapse after surgery, nevertheless multiple retrospective studies show that most patients with large tumors do not have recurrent disease or liver metastases after resection, and are histologically “benign” grade 1 tumors without invasion or involved lymph nodes. It is likely that at least a subgroup of these patients could benefit from conservative therapy, as their tumors might actually be indolent, despite the large tumor size. Additional markers to identify this subgroup are necessary to reduce potential over‐treatment of these patients. Conversely, preoperative markers to identify the aggressive PanNETs of smaller size could be useful to identify patients that require earlier surgery instead of “wait and scan”.

Several markers have the potential to identify high and low risk subgroups. PanNET tumorigenesis is commonly driven by mutations in the tumor suppressor genes *MEN1*, *ATRX*, or *DAXX*, of which the latter two are associated with the alternative lengthening of telomeres phenotype (ALT) and correlate with worse prognosis and liver metastases.^4^, ^5^, ^6^, ^7^, ^8^, ^9^ Protein loss, detected by immunohistochemistry (IHC), can be used as a surrogate for somatic inactivating mutations in *ATRX* and *DAXX*.^9^, ^10^ Recently, promising results in determining these markers in preoperative cytologic specimens have been published.^11^ Nevertheless ATRX/DAXX IHC poses some difficulties, such as the need for positive internal controls, intratumoral heterogeneity, and presence of mutations (eg, missense) that alter function, but not protein expression or localization. Although assessment of ALT is more reliable, nearly 50% of metastatic PanNETs do not have ATRX/DAXX alterations or ALT, thereby emphasizing the need for additional predictive biomarkers.^5^

Recent studies demonstrate that most PanNETs fall into two major subtypes, based on H3K27ac and H3K4me2 signatures, which resemble either normal islet alpha or beta cells. As a surrogate marker of these subtypes, endocrine transcription factors ARX (alpha cell) and PDX1 (beta cell) can be used, and more than half of functional and non‐functional PanNETs showed mutually exclusive expression of these transcription factors. Intriguingly, relapse was almost exclusively associated ARX positive, PDX1 negative or double negative group. PDX1 or double positive expression on the other hand identified a more indolent group, in which only few tumors relapsed.^12^ Similarly, Chan et al showed, using RNA sequencing and whole genome methylation, that the ARX positive, PDX1 negative subgroup was often associated with somatic mutations in *ATRX*, *DAXX*, or *MEN1* and a worse prognosis.^13^

Thus, ARX and PDX1, in combination with ALT, may be prognostic markers to identify low and high risk subgroups preoperatively on cytology, as staining of these proteins seems to identify these alpha and beta cell‐like subgroups robustly. Although, a prerequisite to be able to consider these markers for routine clinical use, is to determine if cytologic material can be reliably used to detect tumor subtype. In addition, inter‐observer agreement between pathologists must be high and methodology for sampling by the gastroenterologist must be established and reproducible. This study aims to answer these questions and provide the framework for further optimization of ARX and PDX1 staining in combination with ALT as preoperative markers, thereby justifying independent validation of these markers in large, prospective trials.

## 2 METHODS

### 2.1 Patient materials

This study was approved by the UMC Utrecht Biobank Research Ethics Committee. The pathology archives were searched for cytology paraffin blocks and corresponding surgical specimens with the diagnosis neuroendocrine tumor/atypical cells of the pancreas. If paraffin blocks were available, the presence of tumor material was confirmed by a H&E stained slide. Data was collected from the pathology report (age, gender, macroscopic size, grade, lymph nodes, margins, and type of paraffin block) and patient files (hormone production, genetic syndromes, endoscopic ultrasound aspiration or biopsy, tumor size, follow‐up, and development of liver metastases).

### 2.2 IHC and fluorescence in situ hybridization

Consecutive 4 μm sections of formalin‐fixed paraffin embedded tissue per case were cleared for 10 minutes at 60°C and deparaffinized in xylene. Endogenous peroxidase was blocked by immersion in 0.6% H_2_O_2_ (Merck 7210, Kenilworth, New Jersey) in methanol for 15 minutes. Antigen retrieval was performed by boiling slides in a 10 mM citrate solution (pH 6) for 20 minutes or a 10/1 mM Tris/EDTA solution (pH 9) in the case of synaptophysin. Nonspecific binding was reduced by blocking with Protein Block Serum Free (Dako, Santa Clara, California, X0909,). ARX (Millipore Burlington, Massachusetts, MABN102, clone 11F6.2, 1:2000), PDX1 (Abcam, Cambridge, United Kingdom, AB134150, clone EPR 3358 (2), 1:2000), Synaptophysin (Novocastra, Amsterdam, The Netherlands, NCL‐L‐SYNAP‐299, clone 27G12, 1:100) and Ki67 (Thermo Fisher, Massachusetts, SP6, Lot. 9106S1805D, 1:100) antibodies were diluted in Normal Antibody Diluent (Immunologic, Duiven, The Netherlands) and incubated for 60 minutes at room temperature. After post antibody blocking (Immunologic) for 15 minutes, the secondary antibody Poly‐HRP‐goat anti Mouse/Rabbit IgG (Immunologic, Cat. no.VWRKDPVB110HRP) was incubated for 30 minutes. Peroxidase activity was detected by DAB (Sigma, St. Louis, Missouri, D5637) or Bright‐DAB for Ki67 slides (cat. no. VWRKBS04‐110, Immunologic) as chromogen for 8 minutes. Slides were shortly counterstained with hematoxylin and coverslips were mounted with Pertex (Histolab, Askim, Sweden).

The telomere‐specific fluorescence in situ hybridization (FISH) was performed as previously described.^14^ Briefly, cleared and deparaffinized sections were boiled in 10 mM citrate solution (pH 6) for 20 minutes. Slides were washed, dried, and the telomere probe (TelC Cy3, F1002 Lot no. 180723PL‐01, 100 nM, Panagene, Yuseong‐gu, Republic of Korea) and the hybridization control centromere probe (Cent‐FITC F3013 Lot No. 172865, 100 nM, Panagene) diluted in hybridization mix (50% deionized Formamide, 50% SCC 4x, 5% Dextran sulfate, Tween‐20 0.5%) were added. Slides were covered and after 5 minutes denaturing at 84°C, slides were quickly cooled and hybridized overnight at 37°C. The next day, slides were washed in two cycles of three times PBS and one time PNA wash buffer (70% Formamide, 30% dH2O, 10 mM Tris). Slides were then counterstained with DAPI in PBS (2 μg/mL, Sigma‐Aldrich, D9542). Slides were washed with dH20, dried and mounted with Vectashield (H‐1000, Vector laboratories, Amsterdam, The Netherlands) and cooled before imaging. Imaging was done with a DM5500 B epifluorescence microscope (Leica Microsystems, Rijswijk, The Netherlands). Photos were made with Leica application Suite X with a Z stack of 14 steps.

All scoring was performed in a blinded fashion for clinical or case specific information. Areas with clumped synaptophysin positive cells in the cytology were annotated for scoring of Ki67, ARX, and PDX1 expression and for assessment of telomere FISH. ARX and PDX1 IHC scoring was performed by two independent researchers (WMH, a medical doctor with more than 4 years of experience in PanNET research, and LAAB, a gastrointestinal pathologist with 10 years of experience in NET diagnostics and research) blinded for each other's results. Both researchers had previous experience with scoring ARX and PDX1 on whole tumor and tissue microarray specimens. Disagreements (if present) were resolved by consensus. Negative protein expression in tumor tissue was defined as weak nuclear staining of ARX or PDX1 in <50% of cells or strong nuclear staining in <10% of cells. Positive ARX or PDX1 expression was called if weak‐nuclear staining was present in >50% of cells or strong nuclear staining >10% of cells as previously described.

The Ki67 labeling index was counted in at least 2000 cells for the surgical specimens by digital image analysis with Sectra (PACS, Sectra AB, Linköping, Sweden) in the areas with highest labeling as previously described.^15^ For the cytologic specimens, all cells in the synaptophysin labeled areas were digitally counted. All digital counts were visually confirmed. Grading was performed following 2017 WHO classification based on Ki67 proliferation index.^16^ If the pathology report mentioned higher tumor grade than the Ki67 count for the surgical specimens, the highest grade was used for the tumor characteristics and dichotomous comparisons with cytology, as higher labeling areas could have been missed in the tested slides. For the continuous comparison between surgical specimens and cytology, the digitally measured Ki67 labeling index was used.

ALT positivity was defined as ultra‐bright, intranuclear telomere FISH signals, more than ×10 the signal intensity of combined telomeres of normal stromal/endothelial cells, if present in >1% of cells determined by visual assessment at ×20 (WMH).^9^ For the surgical specimens, ×100 magnification grayscale images were obtained of areas with suspected ultrabright foci surrounded by stromal cells, and telomere signals were quantified and confirmed using Telometer (a free custom software ImageJ plug‐in, downloaded from http://demarzolab.pathology.jhmi.edu/telometer) and analyzed as previously described. For the cytologic specimens, ultrabright foci were not confirmed with Telometer due to common high background fluorescence. Centromeric FISH signals were used to exclude nonspecific foci and as positive hybridization control. If less than 100 cells could be counted in the cytology by the automated Ki67 count or no ultrabright foci were observed, cases were called non‐informative.

### 2.3 Statistics

Data was analyzed in SPSS version 25 (IBM Nederland, Amsterdam, The Netherlands). To determine inter‐observer agreement, the percentage of agreement, and the Kappa value were calculated. For comparisons between matched samples (cytologic and surgical specimens) the percentage of concordance, sensitivity, specificity, and the Kappa value were calculated. To assess prognostic value, dichotomous comparisons were analyzed using Fisher's exact test. Continuous comparisons like Ki67 labeling index were made using the Wilcoxon signed rank test.^17^

## 3 RESULTS

### 3.1 Patient and material characteristics

Endoscopic fine‐needle aspiration cellblock material from 20 patients with a PanNET diagnosis, 13 of which had corresponding surgical specimens, was included (Table 1). Fourteen cytologic specimens were formalin fixed, agar‐gelatin, or paraffin embedded blocks, the remainder was fixed with Cytolyt and subsequently with formalin. One case was Cytolyt fixed and embedded as Cellient (paraffin) block. All surgical specimens were FFPE blocks.

**Table 1:** Patient characteristics

	All cases
Included cases: H&E cytology representative, n (%)	20 (100)
*Formalin‐fixed paraffin‐embedded*	2
*Formalin‐fixed agar‐gelatin paraffin‐embedded*	12
*CytoLyt‐fixed and formalin‐fixed paraffin‐embedded*	5
*CytoLyt‐fixed Cellient*	1
Male gender, n (%)	13 (65)
Age ± SD (y)	52.9 (14.8)
Non‐functional PanNET, n (%)	18 (90)
Insulinoma, n	2
Sporadic, n (%)	16 (80)
MEN1, n	2
TSC, n	2
EUS FNA, n (%)	15 (75)
EUS FNB, n	1
EUS not specified, n	4
Location Head, n (%)	5 (25)
Location Body, n (%)	3 (15)
Location Tail, n (%)	12 (60)
Resection primary tumor, n	13
Size ± SD (cm)	5.2 (6.2)
T1, n (% of resected)	8 (62)
T2, n (% of resected)	5 (38)
Radical, n (%)	9 (69)
+ Lymph nodes, n (%)	5 (38)
− Lymph nodes, n (%)	6 (46)
No nodes reported, n	2 (15)
No resection	7
Size ± SD (cm)	1.7 (0.7)
Liver metastasis, n (%)	5 (25)

Abbreviations: EUS, endoscopic ultrasound; FNA/B, fine‐needle aspiration/biopsy; H&E, Hematoxylin and Eosin stained; MEN1, multiple endocrine neoplasia 1; SD, standard deviation; TSC, tuberous sclerosis complex.

### 3.2 Cytologic vs surgical specimens

**Figure 1:**
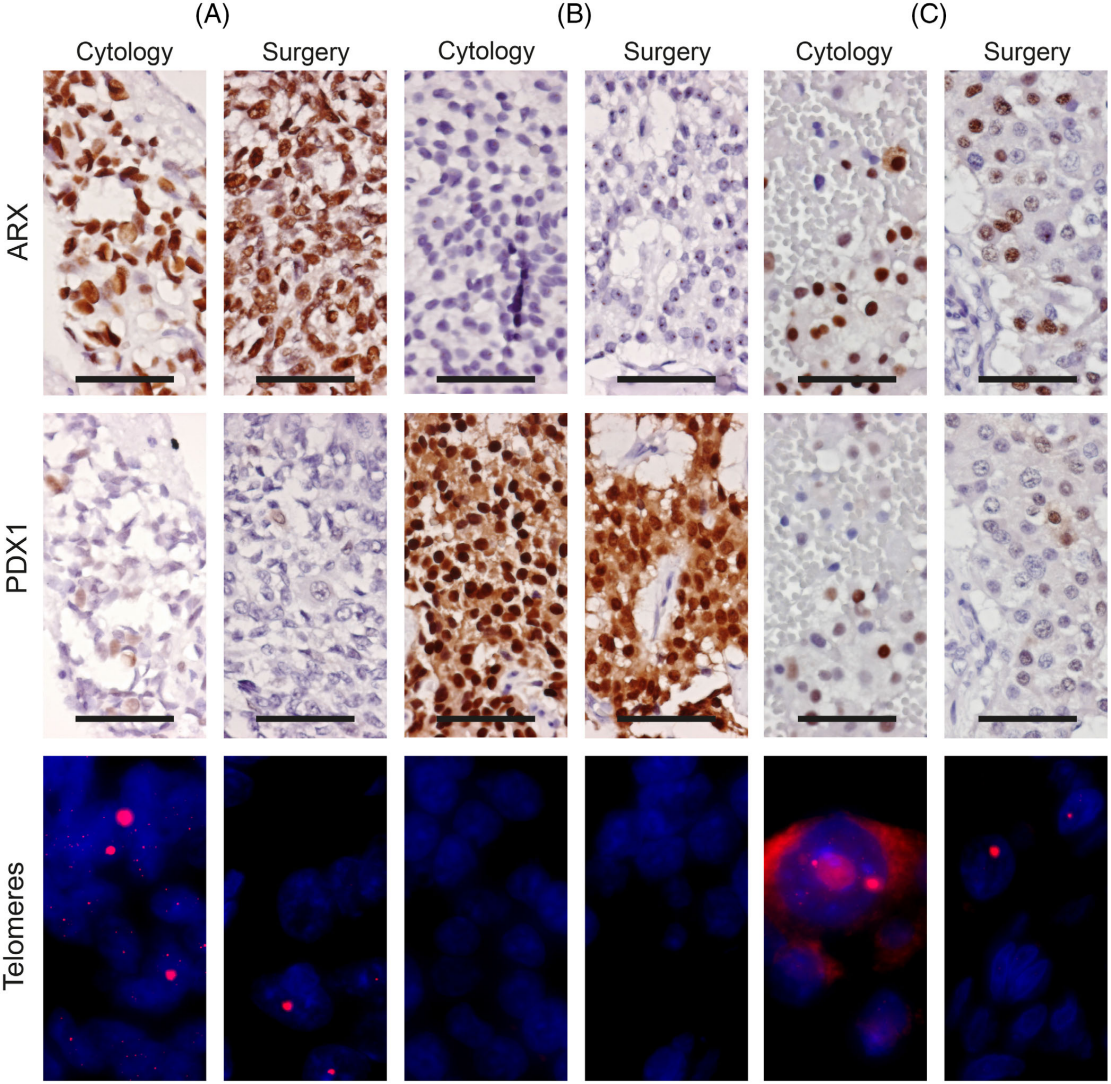
Immunohistochemistry and telomere‐specific fluorescence in situ hybridization of cytologic and surgical specimens. Representative images of cytologic and surgical specimens. A, patient 13; ARX positive, PDX1 negative tumor, ALT positive. B, patient 6; ARX negative, PDX1 positive tumor, without ALT. C, patient 10, discordant case for PDX1 expression; ARX positive, PDX1 negative, ALT positive surgical specimen. PDX1 positive cells are present in the surgical specimen but are below the defined cut‐off. Also note the cytoplasmic background fluorescence in the telomere FISH cytologic specimen. IHC at ×40, 50 μm scale bar. Telomere FISH at ×100, nucleus visible as DAPI blue and ultrabright telomeric signals in the red channel [Color figure can be viewed at http://wileyonlinelibrary.com]

Overall, using a representative surgical specimen tumor block as reference standard, 85% of the cytology cases (11/13 correct) were accurate in determining tumor transcription factor subtype (ARX+/PDX1−, ARX−/PDX1+, ARX+/PDX1+, ARX−/PDX1−) (Figure 1). Concordance of cytologic and surgical specimens for ARX and PDX1 IHC, ALT status, and WHO tumor grade was 100% (Kappa = 1), 85% (Kappa = 0.70), 91% (Kappa = 0.82), and 73% (Kappa = 0.29), while the sensitivity and specificity were 100%/100%, 100%/75%, 83%/100%, and 25%/100%, respectively (Table 2). Two ARX+/PDX1− cases in the surgical specimens were scored as ARX+/PDX1+ in the cytology specimens. For these cases, additional tumor blocks from the surgical specimen were stained to exclude intratumoral heterogeneity often seen in larger tumors which could explain the discrepancy, but the size was average (3.0 and 3.6 cm) and no areas of demarcated heterogeneous expression were noted. Both surgical specimens approached the defined cut‐offs for positive expression. Focal areas of more than 50% of weakly positive cells were found, as well as areas with few strong positive cells, however, these fell just below the defined cut‐offs for the whole slide (Figure 1C). Also, one PDX1 negative surgical specimen was surrounded by stroma with many strong positive PDX1 positive islets of Langerhans (which would have been synaptophysin positive in the cytology). In both discrepant cases, more than 500 cells were present in the cytologic specimens. In one case (patient 11), both observers noted heterogeneous (patchy) positive PDX1 expression in the primary tumor, which was also independently noted when scoring the corresponding cytologic specimen. All other cases showed homogeneous protein expression throughout the tumor.

**Table 2:** Kappa coefficients of comparison surgical and cytologic specimens for prognostic markers

Surgical specimen									
Cytologic specimen		PDX1 +	PDX1 −	Sensitivity	Specificity	Accuracy	Kappa	SE of Kappa and 95% Confidence interval	Strength of agreement
	PDX1 +	5	2	100%	75%	84.6%	0.698	0.187 and 0.330 to 1.000	Good
	PDX1 −	0	6						
								
		ARX +	ARX −						
	ARX +	11	0	100%	100%	100%	1.000	0 and 1.000 to 1.000	Perfect
	ARX −	0	2						
								
		ALT +	ALT −						
	ALT +	5	0	83.3%	100%	90.9%	0.820	0.169 and 0.488 to 1.000	Very good
	ALT −	1	5						
								
		WHO grade 2	WHO grade 1						
	WHO grade 2	1	0	25%	100%	72.73%	0.286	0.241 and 0.188 to 0.759	Fair
	WHO grade 1	3	6						

*Notes*: Two cases excluded (not interpretable) for ALT and grade.

**Figure 2:**
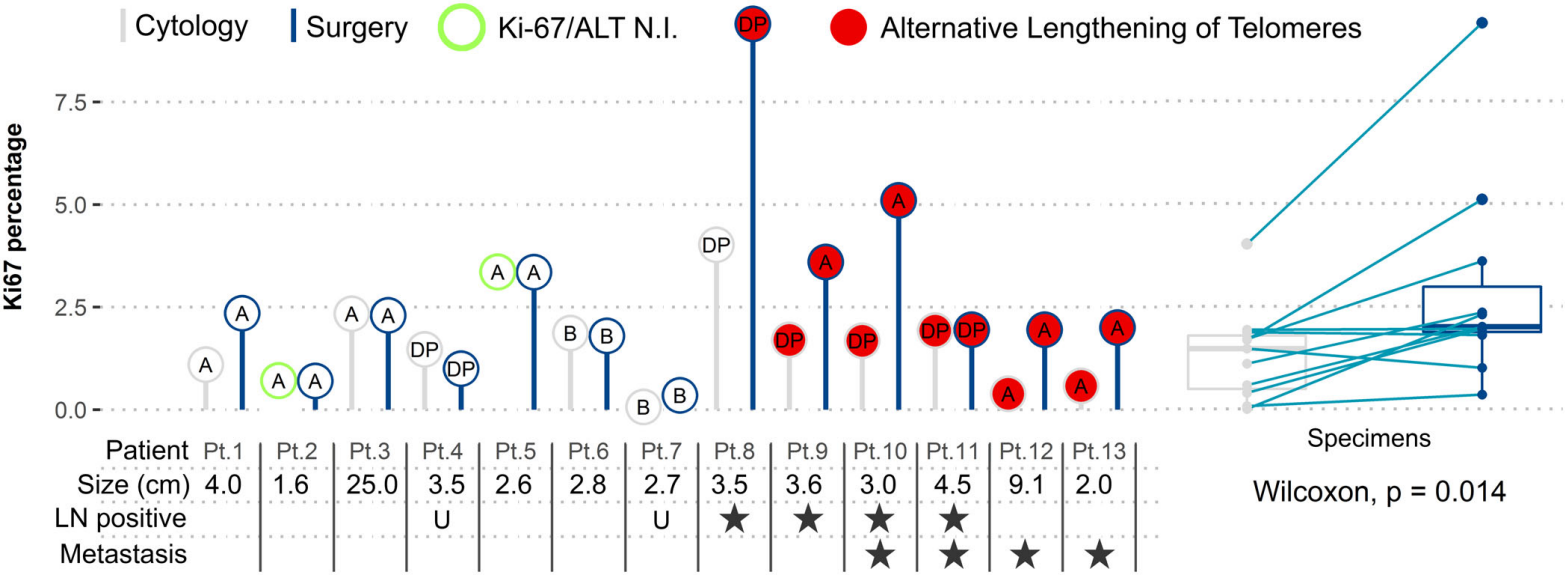
Comparison of matched cytologic and surgical specimens. Thirteen patients for which the cytologic specimen (gray bar and circle) was compared to the surgical specimen (blue bar and circle). Differences in transcription factor subtype (text within circle), telomere phenotype (circle fill), and Ki67 labeling index (y‐axis) can be observed between cytologic and surgical specimens. For patients 2 and 5, the telomere phenotype and Ki67 index were not interpretable. Primary tumor size and behavioral characteristics are given per patient. Comparison of continuous Ki67 labeling index is shown on the right side with a paired Wilcoxon test. A, ARX positive; B, PDX1 positive; DP, double (ARX/PDX1) positive; LN, lymph nodes; N.I., not interpretable; U, unknown [Color figure can be viewed at http://wileyonlinelibrary.com]

One case with ALT was missed by cytology. For this case 498 cells were counted in the cytology. Although one possible ultrabright focus was found in this cytologic specimen, it was below the 1% cutoff. Only one out of four WHO grade 2 tumors were correctly identified by cytology, the continuous Ki67 labeling index in cytology significantly underestimated the percentage of Ki67 positive cells compared to the surgical specimen. (Wilcoxon *P* = .014, Figure 2). Concordance was not better in smaller tumors or cytologic specimens with higher cell counts.

### 3.3 Cytology methods, inter‐observer agreement

There were no clear variations in immunogenicity for IHC observed between different fixatives, FNA or FNB. Both discrepant cases were agar FFPE (2/13). For the single Cellient block case, the Ki67 percentage count failed due to loss of morphology and lack of cell clusters, and the telomere FISH was not interpretable due to hybridization failure. There was perfect inter‐observer agreement in scoring the cytology cases and the primary tumors for ARX and PDX1 (Kappa 1).

### 3.4 Clinical behavior

Of 20 patients, 5 developed liver metastases during follow‐up (Table S1). Marker expression was preferably determined in the surgical specimens, but in cytologic specimens if surgery was not performed. All metastatic tumors expressed ARX, two also expressed PDX1 (double positive). In the non‐metastatic tumors 12 out of 15 had ARX expression of which 2 were double positive, while 2 tumors had sole PDX1 expression and 1 tumor had no ARX or PDX1 expression (double negative) (Table S1). Mean size was not significantly different in metastatic vs non‐metastatic cases (4.3 vs 3.9 cm). Only ALT positivity was significantly associated with liver metastases (Fisher's exact *P* = .0014).

## 4 DISCUSSION

ARX expression was found to be perfectly concordant between preoperative cytologic specimens and postoperative surgical specimens and there was no inter‐observer variation. ALT was confirmed to be a reliable marker with very good concordance between cytologic and surgical specimens.^11^ Furthermore, the presence of ALT was the best predictor of metastatic tumor behavior. Most cytologic specimens were correctly classified as normal or ALT, but one ALT positive case was missed. PDX1 showed a fair concordance between cytologic and surgical specimens, but did not identify indolent tumors in this small cohort.^12^ PDX1 expression has been described before in the metastatic setting.^18^ Furthermore, in contrast to ARX, PDX1 is expressed in pancreatic acinar and ductal cells (Figure S1), and more abundantly in duodenum and islets of Langerhans, causing potential false‐positive PDX1 staining due to contamination—a possible explanation for the discrepancies in this study. Also, focal variation in staining intensity and percentage together with scoring cut‐offs make reliable interpretation of PDX1 on cytology more prone to error. Since reliable cytologic determination of PDX1 is critical to determine the prognostic subgroups as described in Cejas et al, cytologic PDX1 interpretation should be performed with caution, but concerning prognostic value, this study was too limited in size to draw strong conclusions. The high percentage of ARX positive non‐functional PanNETs (17/18) compared to 50% to 60% in recent studies,^12^, ^13^ might be caused by selection bias and reflect more aggressive/faster growing tumors that warranted biopsy.

Ki67 count in cytologic specimens generally underestimated Ki67 count as seen in corresponding surgical specimens (Figure 2). This led to three grade 2 tumors to be erroneously classified as grade 1 tumors based on the Ki67 count in the cytologic specimen. Difficulties of correctly diagnosing intermediate grade or grade 2 PanNETs in cytology have often been reported and usually lead to an underestimation of grade.^19^, ^20^, ^21^, ^22^, ^23^, ^24^, ^25^ It has been shown that accuracy decreases with larger tumors (with possible heterogeneity) and paucicellular cytologic specimens, but this was not seen in the current study.^21^, ^24^, ^26^, ^27^

The use of synaptophysin stained slides, annotated for areas with clusters of NET cells proved to be very useful and time saving in maximizing the cell count from cytologic specimens, without losing specificity (eg, lymphocytes or reactive tissue can have Ki67 positivity, and necrotic debris and blood often have nonspecific binding fluorescent foci which need to be manually check to be excluded). Theoretically, a multiplex/double synaptophysin immunofluorescence staining and telomere FISH would have been even more accurate, but this was not feasible due to intense (cytoplasmic) background fluorescence often observed in the cytologic specimens (Figure 1C). Nevertheless, our methodology of using consecutive slides was sufficient as the clusters of cells were always identified exactly as on the annotated slides.

While 12/13 cases were concordant between cytology and the resection for ALT status, one ALT positive case was missed on cytology, which can be explained by the definition of the cut‐off to call ALT (>1%). In fact, one ultrabright focus was observed in this false‐negative cytologic specimen (1/498), while not a single focus was found in any of the cytologic specimens of the true ALT negative cases. It might therefore be better to use a higher cut‐off (eg, 1000 cells) to determine if a sample has normal telomeres instead of the 100 cells used in this study, or to call ALT positivity in case of any focus is seen in cytology, irrespective of percentage, as these foci appear to be very specific for ALT in cytology.

To conclude, ALT (detected by telomere FISH) and ARX protein expression (detected by IHC) can reliably be determined in cytologic specimens, irrespective of aspiration or most fixation methods. In contrast, PDX1 is less attractive for preoperative use. Ki67 count in cytology frequently underestimates tumor grade and should therefore be interpreted with great caution. Further validation of ARX and PDX1 protein expression in larger retrospective cohorts should be performed to definitively determine their true prognostic value while prospective preoperative validation of ALT as a biomarker in PanNET is warranted.

## CONFLICT OF INTEREST

None

## Supporting information


**Figure S1** Expression of ARX and PDX1 in tissue types encountered during EUS biopsies of PanNETs
Pancreas: PDX1 is expressed strongly in the nucleus and faintly in the cytoplasm of most pancreatic islet cells. In the exocrine compartment, nuclear PDX1 expression can be observed in the centroacinar and ductal cells. ARX is expressed only in a peripherally located subset of islet cells and is completely absent in the exocrine compartment. Duodenum: PDX1 is expressed in duodenal epithelial cells including the absorptive and enteroendocrine cells, but not the goblet cells and submucosa or lymphocytes. Mucous cells in Brunner's glands also express PDX1 strongly. ARX expression can be observed in few cells migrating along the villi, most likely enteroendocrine cells. Brunner's glands and all other cells do not show ARX protein expression. Stomach: Few cells in the gastric mucosa show moderate to strong expression of ARX and PDX1 and can only be found in the glandular compartment. The foveolar cells generally do not express PDX1, but in one case expression was observed in normal foveolar epithelium surrounding gastric intestinal metaplasia. Photos made at ×10, scale bar 250 μm, with ARX and PDX1 IHC and hematoxylin counterstaining

**Table S1** Case and sample immunohistochemical and clinicopathological characteristics
