# Supplementary figures and images for: Assessment of ARX expression, a novel biomarker for metastatic risk in pancreatic neuroendocrine tumors, in endoscopic ultrasound fine‐needle aspiration
Source: Diagn Cytopathol. 2019 Dec 17;48(4):308–15. doi: 10.1002/dc.24368 (PMC7079001; doi:10.1002/dc.24368)

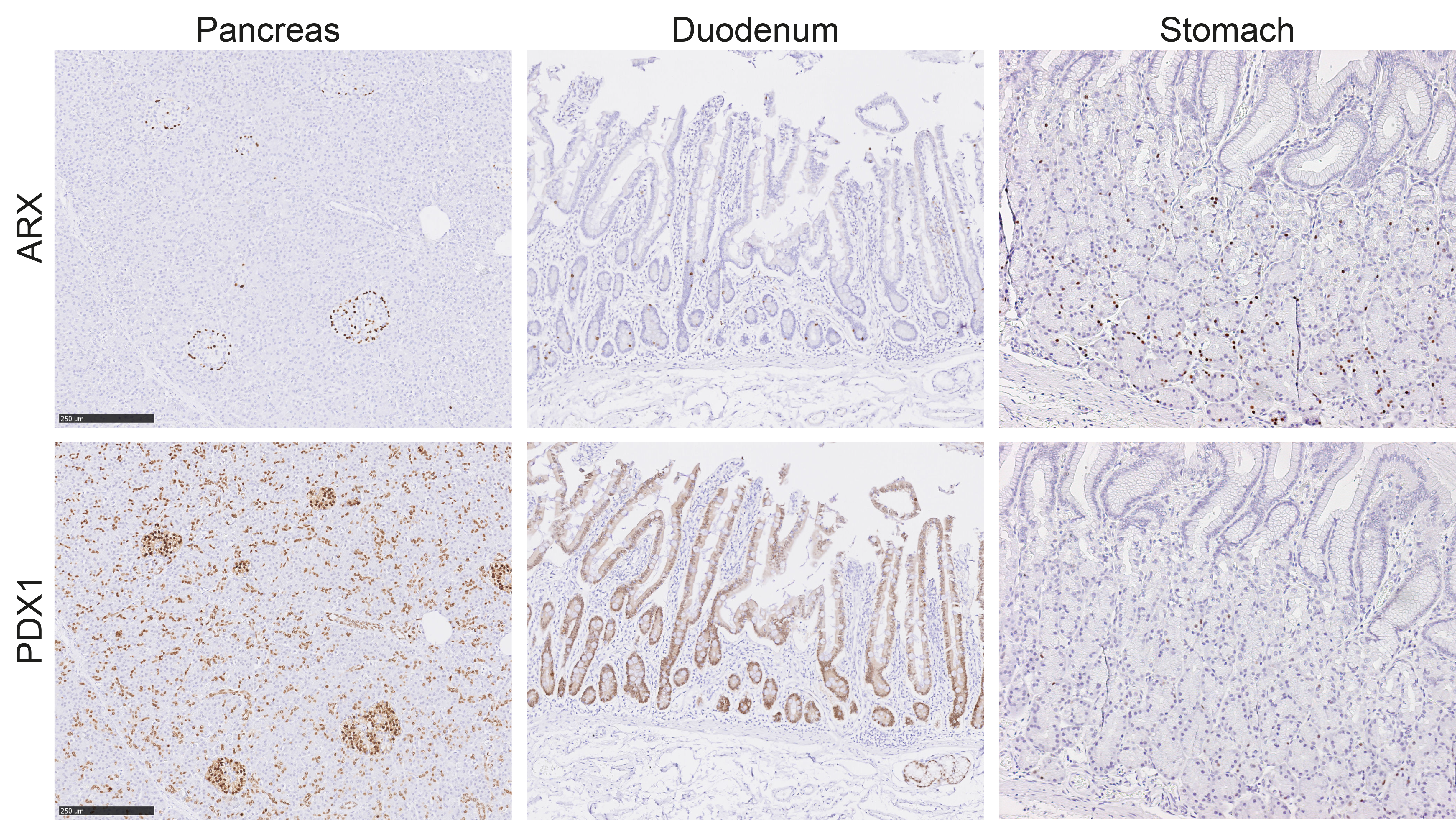

Supplement: Supplementary file 1 — Figure S1 Expression of ARX and PDX1 in tissue types encountered during EUS biopsies of PanNETs Pancreas: PDX1 is expressed strongly in the nucleus and faintly in the cytoplasm of most pancreatic islet cells. In the exocrine compartment, nuclear PDX1 expression can be observed in the centroacinar and ductal cells. ARX is expressed only in a peripherally located subset of islet cells and is completely absent in the exocrine compartment. Duodenum: PDX1 is expressed in duodenal epithelial cells including the absorptive and enteroendocrine cells, but not the goblet cells and submucosa or lymphocytes. Mucous cells in Brunner's glands also express PDX1 strongly. ARX expression can be observed in few cells migrating along the villi, most likely enteroendocrine cells. Brunner's glands and all other cells do not show ARX protein expression. Stomach: Few cells in the gastric mucosa show moderate to strong expression of ARX and PDX1 and can only be found in the glandular compartment. The foveolar cells generally do not express PDX1, but in one case expression was observed in normal foveolar epithelium surrounding gastric intestinal metaplasia. Photos made at ×10, scale bar 250 μm, with ARX and PDX1 IHC and hematoxylin counterstaining [file DC-48-308-s001.tif]
