# Supplementary material for: Assessment of ARX expression, a novel biomarker for metastatic risk in pancreatic neuroendocrine tumors, in endoscopic ultrasound fine‐needle aspiration
Source: Diagn Cytopathol. 2019 Dec 17;48(4):308–15. doi: 10.1002/dc.24368 (PMC7079001; doi:10.1002/dc.24368)
Supplement: Supplementary file 2 — Table S1 Case and sample immunohistochemical and clinicopathological characteristics [file DC-48-308-s002.docx]

**Supplementary Table 1. Case and sample immunohistochemical and clinicopathological characteristics**

| Patient  (Fig 2) | Age  (year) | Sex | Type of tumor | Size  (cm) | ALT Cytology | ALT Surgical | Subtype Cytology | Subtype Surgical | Grade Cytology | Grade Surgical | Cells counted in cytology | FU from surgery | Liver metastases | Lymph node positive |
| --- | --- | --- | --- | --- | --- | --- | --- | --- | --- | --- | --- | --- | --- | --- |
| 1 | 61 | F | NF | 4 | normal | normal | A | A | 1 | 1 | 1977 | 104 | no | no |
| 2 | 21 | F | NF | 1.6 | NI (few cells) | normal | A | A | NI | 1 | 4 | 61 | no | no |
| 3 | 46 | M | NF | 25 | normal | Normal | A | A | 1 | 1 | 1578 | 13 | no | Yes |
| 4 | 44 | F | Ins | 3.5 | normal | Normal | DP | DP | 1 | 1 | 136 | 19 | no | Not reported |
| 5 | 19 | M | NF | 2.6 | NI (Cellient) | Normal | A | A | NI | 2 | NI | 38 | no | no |
| 6 | 65 | M | NF | 2.8 | normal | Normal | B | B | 1 | 1 | 2949 | 12 | no | no |
| 7 | 50 | M | Ins | 2.7 | normal | Normal | B | B | 1 | 1 | 2741 | 52 | no | Not reported |
| 8 | 62 | M | NF | 3.5 | normal***** | ALT | DP | DP | 2 | 2 | 498 | 9 | no | Yes |
| 9 | 60 | M | NF | 3.6 | ALT | ALT | DP***** | A | 1***** | 2 | 2000 | 13 | no | Yes |
| 10 | 48 | M | NF | 3 | ALT | ALT | DP***** | A | 1***** | 2 | 891 | 8 | Yes | Yes |
| 11 | 74 | M | NF | 4.5 | ALT | ALT | DP | DP | 1 | 1 | 2074 | 0 | Yes | Yes |
| 12 | 69 | F | NF | 9.1 | ALT | ALT | A | A | 1 | 1 | 2554 | 19 | Yes | no |
| 13 | 46 | F | NF | 2 | ALT | ALT | A | A | 1***** | 2 | 695 | 43 | Yes | no |
|  | 67 | M | NF | 1.9 | normal | No surgery | DP | No surgery | NP | No surgery | NP |  | no | No surgery |
|  | 61 | M | NF | 1.6 | normal | No surgery | A | No surgery | NP | No surgery | NP |  | no | No surgery |
|  | 42 | F | NF | 2.1 | normal | External surgery | DP | External surgery | NP | External surgery | NP | unknown | no | Yes |
|  | 71 | M | NF | 1.1 | normal | No surgery | DN | No surgery | 1 | No surgery | 230 |  | no | No surgery |
|  | 50 | M | NF | 0.8 | normal | No surgery | A | No surgery | 1 | No surgery | 447 |  | no | No surgery |
|  | 53 | M | NF | 1.5 | normal | No surgery | A | No surgery | 1 | No surgery | 1680 |  | no | No surgery |
|  | 49 | F | NF | 3 | ALT | External surgery | DP | External surgery | 2 | External surgery | 2835 | 12 | Yes | Not reported |
| ALT, alternative lengthening of telomeres; FU, follow-up; NF, non-functional; Ins, Insulinoma; A, ARX positive; B, PDX1 positive; DP, double (ARX/PDX1) positive; DN, double (ARX/PDX1) negative; NI, not interpretable; NP, not performed. *discrepancy between cytologic and surgical specimens | | | | | | | | | | | | | | |
